# Ventricular restoration in adults with huge congenital left ventricular aneurysm: report of two cases

**DOI:** 10.11604/pamj.2024.48.8.36988

**Published:** 2024-05-06

**Authors:** Charles Mve Mvondo, Hermann Nestor Tsague Kengni, Laurence Carole Ngo Yon, Amalia Ariane Owona Ngandebe, Etienne Sene, Marcelin Ngowe Ngowe

**Affiliations:** 1 Division of Cardiac Surgery, Shisong Cardiac Centre, Kumbo, Cameroon,; 2 Faculty of Medicine and Pharmacy, University of Douala, Douala, Cameroon,; 3 Department of Internal Medicine, University of Yaoundé 1, Yaoundé, Cameroon,; 4 Department of Anesthesia and Intensive Care Unit, Fann University Hospital, Dakar, Senegal

**Keywords:** Congenital aneurysm, ventricular restoration, Cameroon, case report

## Abstract

Congenital ventricular aneurysms (CVA) are rare cardiac anomalies that have been predominantly described in the Black population. They are characterized by an akinetic ventricular protrusion that is commonly located at the basal and apical segments. Although the diagnosis is often incidental and the majority of patients are asymptomatic, life-threatening events such as persistent ventricular arrhythmias, CVA rupture, and heart failure are not uncommon. However, no standardized therapy is currently available and good outcomes have been reported with both conservative and surgical management. We report the cases of two young Black African patients with huge symptomatic CVA lesions who underwent successful surgical repair with a ventricular restoration technique. Both cases were consulted for chest pain and dyspnea. Chest X-ray and transthoracic Doppler echocardiography suggested the diagnosis. Thoracic angioscanner and thoracic magnetic resonance imaging confirmed the diagnosis. Both patients underwent successful surgery. This case report aims to revisit the diagnostic and therapeutic approach to this rare pathology, in our professional environment.

## Introduction

Congenital ventricular aneurysms (CVA) are rare entities with an estimated incidence between 0.02% and 0.34% [1,2]. The CVA appears as a saccular and akinetic extension of the ventricular wall, mainly located at the apical segment in the majority [2]. The left ventricle is the mostly affected, and the differential diagnoses are with congenital ventricular diverticula (CVD) and acquired ventricular aneurysms such as post-myocardial infarction aneurysms and infectious conditions such as viral cardiomyopathy and myocardial involvement in Chagas and tuberculosis diseases [3-6]. Despite the risk of life-threatening complications such as rupture, there has been some reluctance to recommend surgical therapy unless symptoms are present or in cases with associated cardiac lesions. We report the cases of two young adults who underwent ventricular restoration for symptomatic and complicated CVA in our institution. This case report aims to revisit the diagnostic and therapeutic approach to this rare pathology, in our professional environment.

## Patient and observation

### Case 1

**Patient information:** a 27-year-old Black male patient was referred to our department from an outside hospital with a diagnosis of a large apical ventricular pseudo-aneurysm. The patient had no familial history of cardiovascular disease or sudden death.

**Clinical findings:** he complained 3 weeks earlier of recurrent headaches, general body weakness, thoracic compression, and sporadic episodes of palpitations.

**Figure 1:**
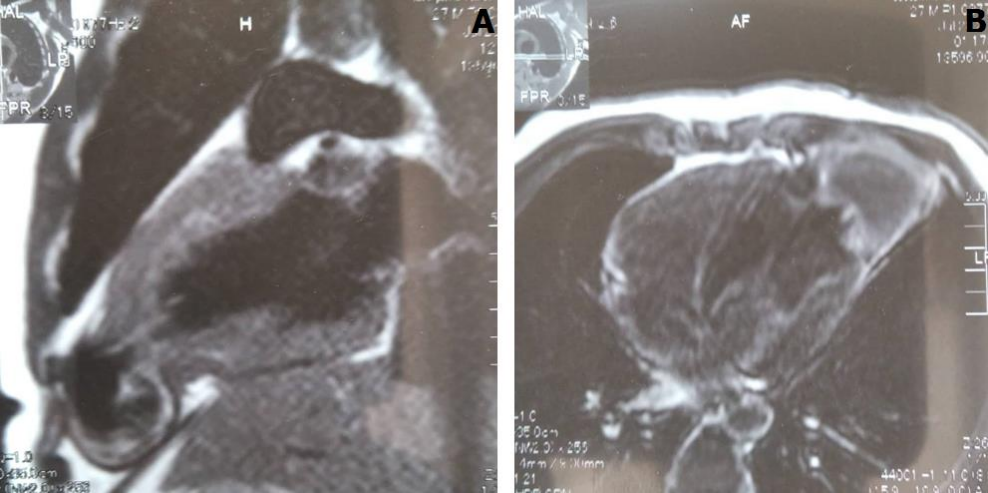
left apical congenital ventricular aneurysms at magnetic resonance imaging (A, B)

**Diagnostic assessment:** the electrocardiogram showed a Cornell index at 28 mm with ST-T changes in leads V5 and V6. While a Color-Doppler transthoracic echocardiogram ruled out valvular, congenital, and ventricular dysfunction, an apical flow acceleration was found with a suspicion of left ventricular diverticulum or aneurysm. The latter was confirmed at subsequent Magnetic Resonance Imaging (MRI) describing an akinetic fibrotic and partially thrombosed cavity, suggesting an apical CVA (Figure 1), communicating with the left ventricle through a 2-centimetre defect. Additional investigations with 24-hour Holter-Electrocardiogram, chest X-ray, and serology (VDRL, HIV) did not reveal other abnormalities.

**Figure 2:**
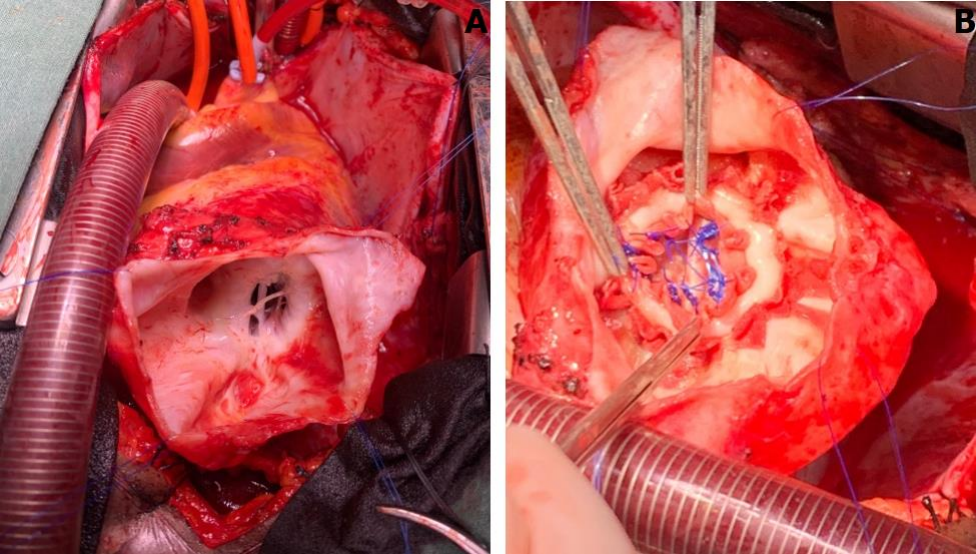
intraoperative views of the congenital ventricular aneurysms, A) apical defect, B) closure of the defect with a bovine pericardium

**Therapeutic interventions:** an oral anticoagulation prophylaxis and beta-blocker therapy were initiated with poor improvement of the symptoms after 3 weeks of treatment. Following a clinical discussion with the following cardiologist and the patient, a consensual decision was taken for elective surgical repair. The patient underwent a successful ventricular restoration with a bovine pericardial patch under cardiopulmonary bypass (Figure 2).

**Follow-up and outcome of interventions:** the postoperative course was uneventful and the patient was discharged from the hospital 6 days after surgery.

**Informed consent:** the patient reported his full consent to publish his case.

### Case 2

**Patient information:** a 29-year-old woman with a known history of thoracic mass was admitted to the emergency department of our institution for progressive tachypnea.

**Clinical findings:** she complained two months earlier of recurrent thoracic compression and dyspnea.

**Figure 3:**
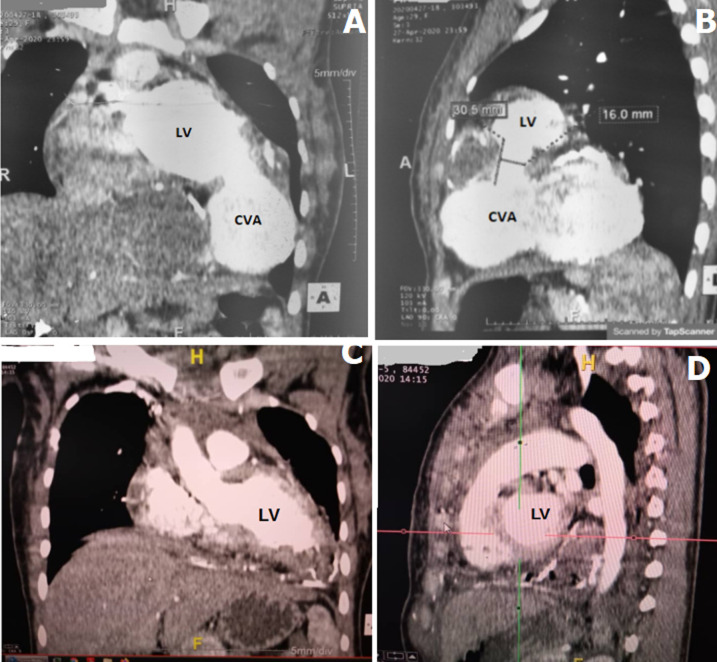
angio-computed tomography scan views: comparative aspects, preoperative (A, B), postoperative (C, D)

**Diagnostic assessment:** a chest X-ray revealed left para-cardiac opacity without parenchymal involvement. The electrocardiogram showed a first-degree atrioventricular block, left ventricular hypertrophy with a Cornell index at 27 mm, Cornell product criteria at 3240mVms, and ST-T changes in leads V5 and V6. An emergency angio-computed tomography scan revealed a huge thrombosed cavity communicating with the left ventricle at the apical segment (Figure 3).

**Figure 4:**
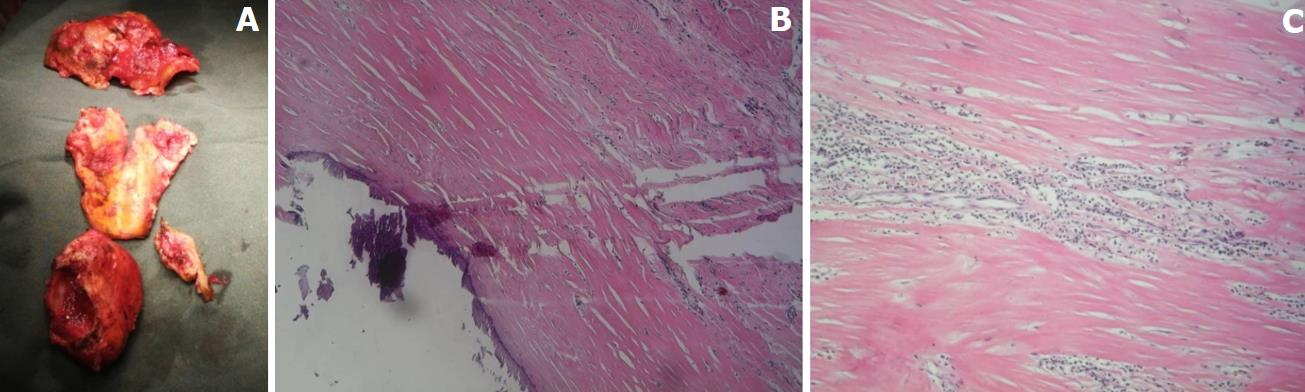
excised congenital ventricular aneurysms with diffused thrombosis (A), histopathology of congenital ventricular aneurysms wall (B, C) showing fibrotic tissue with diffused inflammatory cells and focus of calcification

**Therapeutic interventions:** considering the risk of rupture, an urgent surgery was planned. A diagnosis of large CVA was made intra-operatively and the patient underwent a surgical ventricular restoration and mass resection (Figure 4).

**Follow-up and outcome of interventions:** the postoperative course was uneventful and the patient was discharged home on the 10^th^ postoperative day. A postoperative angio-computed tomography scan showed no residual shunt and good restoration of the left ventricular shape (Figure 3).

**Informed consent:** the patient reported her full consent to publish her case.

## Discussion

CVA lesions are a large spectrum of heterogeneous patterns varying from isolated myocardial protrusions, described mostly in autopsy studies, to voluminous protuberances as observed in the current series. While the precise mechanism of CVA development remains unknown, embryogenic defects within the endocardial tube from the 4^th^ embryogenic week have been hypothesized [7]. No evidence of sex predominance is supported by various reports, while the majority of CVA patients were mainly from African and American regions [2]. Morphologically, CVAs appear as akinetic and fibrous protuberances of the ventricular wall, with a predominant location at the apical segment [1,2]. They are histologically distinct from congenital ventricular diverticula (CVD) which wall presents intrinsic similarities with myocardial tissue, including contractile activity. As opposed to CVA, CVD is commonly associated with cardiac and/or extra-cardiac lesions [8]. Other differential diagnoses are post-infarction ventricular aneurysms and aneurysmal lesions from infectious processes such as the human immunodeficiency virus, Chagas, and tuberculosis diseases [3-6]. The heterogeneity of CVA lesions, with regards to size, location, thrombosis, and associated anomalies, translates in a variety of clinical presentations from asymptomatic status which is the majority, to more invaliding events such as persistent arrhythmias, heart failure, rupture, and sudden death [9].

The diagnosis of CVA is often incidental during routine investigations for other diseases. Although CVA can be associated with electrical abnormalities [10], these events are relatively rare [11]. Pellicia *et al*. suggested a classification in 3 groups (distinct, mild, and minor) of these electrocardiographic abnormalities [12]. According to that classification, the abnormalities observed were then mild although the sensitivity, specificity, positive predictive value, and negative predictive value of a 12-lead ECG for the diagnosis of CVA are low [11]. CVA lesions can be primarily detected by Color-Doppler Echocardiography which is largely accessible and provides a reliable description of ventricular morphology and associated cardiac anomalies, despite misdiagnoses of small apical lesions are possible [13,14]. Complementary imaging with magnetic resonance imaging (MRI), computed tomography scan, and conventional computed angiography is often required in doubtful cases and when surgical correction is considered. They provide specific details on CVA tissue, size, kinesia (to differentiate CVA from CVD), and associated lesions such as congenital anomalies in children and/or coronary disease in adult patients [15,16]. To date, no consensus exists in clinical practice for the management of CVA due to its scarce prevalence. Indeed, limited data from case series have presented heterogeneous outcomes with several strategies including surgical repair, antiarrhythmic treatment, and conservative management among others [2,9]. In a study by Mayer *et al*., no cardiac death was observed among patients with CVA and CVD who underwent conservative management over a 13-year follow-up period [17]. Similar experiences with non-surgical approaches were reported by other authors with imaging follow-up from the fetal period [18,19]. However, surgery was mostly described in cases with associated anomalies or those with clear symptoms, following congestive heart failure, thromboembolism, and sustained ventricular arrhythmias. The type of surgery depends on the size and location of the CVA, in addition to the associated anomalies. Simple direct suturing of small defects (<2 cm) is often sufficient, whereas larger lesions require more complex procedures such as ventricular restoration with circular patches as described in post-infarction ventricular aneurysm surgery [1,20]. Thus, care should be taken to avoid restrictive dysfunction following ventricular repairs, and mitral valve insufficiency resulting from displacement/distortion of the papillary muscles. If the operative risk is relatively low (<2%) in isolated lesions, it might significantly increase in cases with associated anomalies. In our case, the repair of a giant thrombosed CVA with partial rupture required femora-femoral cannulations for cardiopulmonary bypass and moderate hypothermia considering the risk of complete rupture during chest entry. In cases presenting with ventricular sustained arrhythmias, antiarrhythmic treatment including radiofrequency ablation or ICD implantation has been described as lone therapy or in combination with surgery [21,22].

## Conclusion

Fatal complications from CVA could be more common than expected. Thus, an accurate evaluation of ventricular wall abnormalities during routine imaging analysis should be considered in patients presenting with symptoms of heart failure, stroke, and ventricular arrhythmias. Surgical treatment is effective and should be considered in cases refractory to conservative therapy.
